# The complete chloroplast genome of a Chinese endemic poorly known species *Campylotropis grandifolia* (Fabaceae)

**DOI:** 10.1080/23802359.2021.2018950

**Published:** 2022-01-10

**Authors:** Hexiang Duan, Wenxiang Liu, Jing Yang, Xuecan Wu

**Affiliations:** a Yunnan Research Academy of Eco-environmental Sciences, Kunming, China; b Key Laboratory of Biodiversity Conservation in Southwest China, State Forestry Administration, Southwest Forestry University, Kunming, China

**Keywords:** *Campylotropis grandifolia*, chloroplast genome, phylogeny

## Abstract

The first complete chloroplast genome of *Campylotropis grandifolia* Schindl. is reported and characterized in this study. The whole chloroplast genome was 153,213 bp in length with 128 genes, including 81 protein-coding genes, 39 tRNAs, and eight rRNAs. Maximum-likelihood (ML) phylogenetic analysis of 25 legume species strongly supported that *Campylotropis* is most closely related with *Kummerowia* and *Lespedeza*, which is consistent with previous studies.

*Campylotropis* Bunge (Fabaceae) is a member of the tribe Desmodieae (Benth.) Hutch. (Iokawa and Ohashi 2002a). Among this genus, 38 species are distributed in temperate and tropical regions across Asia, and most species are located in the Southwest China, which is the diversity center of this genus (Lokawa and Ohashi 2002a, 2002b, 2008; Huang et al. 2010; Liao and Xu 2020). *Campylotropis grandifolia* Schindl. is known for the quadrangular branches and lack of glandular hairs on the inflorescences (Lokawa and Ohashi 2002b). Since few studies have focused on chloroplast genome of *Campylotropis*, the chloroplast of *C. grandifolia* can help to resolve the intergeneric and intrageneric relationships in legume species.

The fresh leaves of *C. grandifolia* were collected from a wasteland in the campus of Southwest Forestry University, Yunnan Province and the voucher specimens were deposited in the herbarium of Ningbo Botanical Garden (NPH, collection #: Kai-Wen Jiang & Wen-Xiang Liu s. n.). Total genomic DNA was extracted with CTAB method, and the genomic library was prepared and paired-end sequenced at Illumina Hiseq 2500 platform (Illumina, San Diego, CA) in Frasergen (Wuhan, China). After filtering the raw data, clean reads were assembled with GetOrganelle v.1.7.0 software (Jin et al. 2020) and manually corrected with Geneious software. Annotation was conducted with PGA v. (Qu et al. 2019) and OGDRAW (Greiner et al. 2019). Finally, the chloroplast genome was deposited in NCBI with the accession of MZ918589.

The total length of chloroplast genome of *C. grandifolia* was 153,213 bp, the total GC content is 35% the specific length and the GC content of each part is shown in Table 1. A total of 111 species and 128 genes were annotated in *C. grandifolia* as shown in Table 2.

**Table 1.** Base composition of chloroplast genome in *Campylotropis grandifolia*.

Species	Genome length (bp)				GC content (%)			
	Total	LSC	SSC	IR	Total	LSC	SSC	IR
*Campylotropis grandifolia*	153213	84739	27678	40796	35.0	30.4	33.3	42.8

IR: inverted repeat; LSC: large single copy; SSC: small single copy.

**Table 2.** Genes present in chloroplast genome of *Campylotropis grandifolia*.

Gene classification	Gene group	The name of the gene
Gene related to photosynthesis	Photosystem I	*psa*A *psa*B *psa*C *psa*I *psa*J
	Photosystem II	*psb*A *psb*B *psb*C *psb*D *psb*E *psb*F *psb*H *psb*I *psb*J *psb*K *psb*L *psb*M *psb*N *psb*T *psb*Z
	Cytochrome b/f complex	*pet*A *pet*B *pet*D *pet*G *pet*L *pet*N
	ATP synthase	*atp*A *atp*B *atp*E *atp*F *atp*H *atp*I
	NADH dehydrogenase	*ndh*A *ndh*B *ndh*C *ndh*D *ndh*E *ndh*F *ndh*G *ndh*H *ndh*I *ndh*J *ndh*K
	RubisCO large subunit	*rbc*L
Expression of related genes	Ribosomal proteins (LSU)	*rpl2 rpl14 rpl16 rpl20 rpl23 rpl32 rpl33 rpl36*
	Ribosomal proteins (SSU)	*rps*2 *rps*3 *rps*4 *rps*7 *rps*8 *rps*11 *rps*12 *rps*14 *rps*15 *rps*16 *rps*18 *rps*19
	Transfer RNAs	*trnA-UGC trnC-GCA trnD-GUC trnE-UUC trnF-GAA trnfM-CAU trnG-GCC trnG-UCC trnH-GUG trnI-CAU trnI-GAU trnK-UUU* *trnL-CAA trnL-UAA trnL-UAG trnM-CAU trnN-GUU trnP-UGG trnQ-UUG trnR-ACG trnR-UCU trnS-GCU trnS-GGA trnS-UGA trnT-GGU trnT-UGU trnV-GAC trnV-UAC trnW-CCA trnY-GUA*
	RNA polymerase	*rpo*A *rpo*B *rpo*C1 *rpo*C2
	Ribosomal RNAs	*rrn*4.5 *rrn*5 *rrn*16 *rrn*23
Other genes		*ccs*A *acc*D *cem*A *clp P mat K*
Hypothetical chloroplast reading frames		*ycf*1 *ycf*2 *ycf*3 *ycf*4
Hypothetical chloroplast reading frames		*ycf*1 *ycf*2 *ycf*3

In order to confirm the systematical position of *C. grandifolia*, a total of 24 legume chloroplast genomes were downloaded from GenBank and applied to construct the phylogenetic tree, where *Lysiphyllum binatum* (Blanco) de Wit was selected as the outgroup. Specially, we aligned these 25 cp genomes with MAFFT v.7.481 (Katoh and Standley 2013), eliminated the gaps with MEGA X (Kumar et al. 2018), and constructed maximum-likelihood (ML) tree with IQ-TREE v.1.6.12 (Nguyen et al. 2015) in K3Pu + F+R5 model (Figure 1). Our results showed that *C. grandifolia* is sister to the clade consisting of *Kummerowia striata* (Thunb.) Schindl. and *Lespedeza bicolor* Turcz., which is congruent with previous study using the combined cpDNA fragments data (Xu et al. 2012).

**Figure 1.**
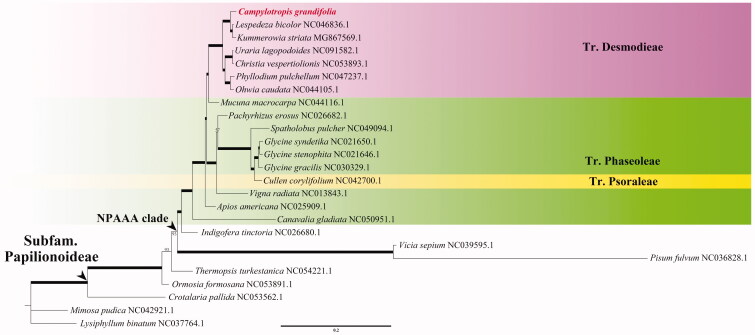
The maximum-likelihood (ML) phylogenetic tree for 25 species of Fabaceae. Bold branches indicate nodes with 100 bootstrap support. The newly sequenced species, *Campylotropis grandifolia* in this study is marked in italic bold font.

## Data Availability

The genome sequence data that support the findings of this study are openly available in The DNA Data Bank of Japan (DDBJ; http://www.ddbj.nig.ac.jp) under the accession no. MZ918589. The associated BioProject and Bio-Sample numbers are PRJNA743046 and SAMN20688300, respectively.
